# Characterization and Immunomodulatory Effects of Canine Adipose Tissue- and Bone Marrow-Derived Mesenchymal Stromal Cells

**DOI:** 10.1371/journal.pone.0167442

**Published:** 2016-12-01

**Authors:** Keith A. Russell, Natalie H. C. Chow, David Dukoff, Thomas W. G. Gibson, Jonathan LaMarre, Dean H. Betts, Thomas G. Koch

**Affiliations:** 1 Departments of Biomedical Sciences, Ontario Veterinary College, University of Guelph, Guelph, Canada; 2 Clinical Studies, Ontario Veterinary College, University of Guelph, Guelph, Canada; 3 Physiology and Pharmacology, The University of Western Ontario, London, Canada; 4 The Orthopaedic Research Lab, Aarhus University, Aarhus, Denmark; Instituto Butantan, BRAZIL

## Abstract

**Background:**

Mesenchymal stromal cells (MSC) hold promise for both cell replacement and immune modulation strategies owing to their progenitor and non-progenitor functions, respectively. Characterization of MSC from different sources is an important and necessary step before clinical use of these cells is widely adopted. Little is known about the biology and function of canine MSC compared to their mouse or human counterparts. This knowledge-gap impedes development of canine evidence-based MSC technologies.

**Hypothesis and Objectives:**

We hypothesized that canine adipose tissue (AT) and bone marrow (BM) MSC (derived from the same dogs) will have similar differentiation and immune modulatory profiles. Our objectives were to evaluate progenitor and non-progenitor functions as well as other characteristics of AT- and BM-MSC including 1) proliferation rate, 2) cell surface marker expression, 3) DNA methylation levels, 4) potential for trilineage differentiation towards osteogenic, adipogenic, and chondrogenic cell fates, and 5) immunomodulatory potency *in vitro*.

**Results:**

1) AT-MSC proliferated at more than double the rate of BM-MSC (population doubling times in days) for passage (P) 2, AT: 1.69, BM: 3.81; P3, AT: 1.80, BM: 4.06; P4, AT: 2.37, BM: 5.34; P5, AT: 3.20, BM: 7.21). 2) Canine MSC, regardless of source, strongly expressed cell surface markers MHC I, CD29, CD44, and CD90, and were negative for MHC II and CD45. They also showed moderate expression of CD8 and CD73 and mild expression of CD14. Minor differences were found in expression of CD4 and CD34. 3) Global DNA methylation levels were significantly lower in BM-MSC compared to AT-MSC. 4) Little difference was found between AT- and BM-MSC in their potential for adipogenesis and osteogenesis. Chondrogenesis was poor to absent for both sources in spite of adding varying levels of bone-morphogenic protein to our standard transforming growth factor (TGF-β3)-based induction medium. 5) Immunomodulatory capacity was equal regardless of cell source when tested in mitogen-stimulated lymphocyte reactions. Priming of MSC with pro-inflammatory factors interferon-gamma and/or tumour necrosis factor did not increase the lymphocyte suppressive properties of the MSC compared to untreated MSC.

**Conclusions/Significance:**

No significant differences were found between AT- and BM-MSC with regard to their immunophenotype, progenitor, and non-progenitor functions. Both MSC populations showed strong adipogenic and osteogenic potential and poor chondrogenic potential. Both significantly suppressed stimulated peripheral blood mononuclear cells. The most significant differences found were the higher isolation success and proliferation rate of AT-MSC, which could be realized as notable benefits of their use over BM-MSC.

## Introduction

Mesenchymal stromal cells (MSC) have progenitor and non-progenitor categories of function that show promise for their clinical use in a wide variety of conditions. Progenitor function refers to the cells' multipotency or their ability to be directed into several cell types including those that make up fat, bone, and cartilage. Non-progenitor function refers to the cells' more recently discovered ability to influence resident cells and tissue functions through their secretome and direct cell-cell contact, including regenerative and immune modulatory effects [1]. These two types of function along with MSC' readiness for *in vitro* expansion have led to much interest from scientists and clinicians alike. Recently, the dog has emerged as an increasingly useful preclinical animal model to study the development and safety of stem cell–based therapies. Comprehensive validation of the utility of canine MSC will provide far-reaching benefit in both the veterinarian field as well as in translational medicine.

The heterogeneity of MSC populations makes definitive characterization inherently challenging. The International Society for Cellular Therapy attempted to simplify this by establishing three criteria to define the MSC: 1) plastic-adherence, 2) specific positive and negative expression of a panel of specific cell surface markers, and 3) trilineage differentiation potential into bone, cartilage, and fat [2]. Unfortunately, while the first criterion is universal enough for cross-species application, the second criterion's surface marker panel is based on human MSC. A corresponding panel for canine MSC is yet to be established, but progress is being made with markers such as CD44 and CD90 showing consistent positive and CD34 and CD45 consistent negative expression [3].

Adipogenesis and osteogenesis are frequently shown in canine MSC studies most often validated with histological staining and sometimes with mRNA expression data of induced versus non-induced MSC populations [3]. Chondrogenic induction of canine MSC has proven challenging using standard protocols and robust chondrogenic differentiation remains to be shown [4–17]. Even in our own previous attempts, we were unable to successfully induce chondrogenesis in our canine cells [18]. However, all of this may be less damaging to the clinical utility of MSC as a paradigm shift directs focus to their non-progenitor functions [19,20].

Early in this century, reports began to emerge of the ability of MSC derived from bone marrow aspirate (BM-MSC) to suppress proliferation of T-lymphocytes after stimulation with allogeneic cells or mitogens [21,22]. Soon after, adipose tissue-derived (AT-)MSC were shown to have similar immunomodulatory properties as their bone marrow-derived counterparts [23]. It has been suggested that MSC effect this immunosuppression through cell-cell contact and secreted soluble factors [24–27]. While some factors are constitutively expressed, others like indoleamine 2,3-dioxygenase (IDO) are induced by pro-inflammatory cytokines such as interferon-gamma (IFN-γ) and tumour necrosis factor-alpha (TNF-α) [28,29]. While activated T-lymphocytes produce IFN-γ and TNF-α, pre-licensing or priming MSC with these inducers in culture promote their immunosuppressive properties [28–32]. Only a few articles on canine MSC immune modulation have been published [33–36], and no comparison of canine AT- and BM-MSC with regards to their immunomodulatory function has been reported, nor have the effects of proinflammatory cytokine-primed canine MSC been studied to date.

In this study, we examined both the progenitor and non-progenitor functions of canine AT- and BM-MSC. Surface marker expression, population doubling times, and DNA methylation quantification were also compared for the purpose of explaining any differences between the cell sources with regards to their differentiation or immunomodulatory capacities.

### Hypothesis

Donor paired canine adipose tissue (AT)- and bone marrow (BM)-derived MSC will have similar differentiation capacity and immune modulatory properties.

### Objectives

To characterize AT- and BM-derived MSC with regards to their:

1. Population doubling time
2. Cell surface marker expression
3. Global DNA methylation quantification
4. Trilineage differentiation potential
5. Immunomodulatory potency

## Materials and Methods

### Ethics statement

Guidelines by the University of Guelph Animal Care Committee were closely followed with regard to the collection of canine blood, adipose tissue, and bone marrow samples. Since collection of these tissue samples occurred post-mortem and dogs were sacrificed for reasons unrelated to the studies, subsequent research conducted using these samples did not require review by the Animal Care Committee (falls under CCAC Category of Invasiveness A). Therefore, these studies were conducted in accordance with the institutional ethics guidelines. Blood and tissues were collected immediately after the dogs were euthanized by intravenous injection of pentobarbital (Euthanyl Forte, 540mg/5 Kg, Biomeda-MTC Animal Health, Cambridge, Ontario) at Hillside Kennels Animal Control, Innerkip, ON. Euthanasia was deemed necessary by the kennel as the dogs were aggressive/dangerous and not suitable for adoption.

### MSC isolation

Cryopreserved AT- and BM-MSC were thawed from previously isolated and cryopreserved cultures from 8 dogs [18]. The dogs used were of unknown age each weighing a minimum 30 kg.

### MSC culture and proliferation

MSC were cultured in expansion medium (EM) composed of low glucose Dulbecco’s modified Eagle’s medium (DMEM, Lonza, Walkersville, Maryland), 10% pooled FBS (Life Technologies, Grand Island, New York), 1% penicillin/streptomycin, and 1% L-glutamine (Lonza, Walkersville, Maryland) and incubated at 38°C in a 5% CO₂ humidified environment. Cells were harvested at 60–80% confluency using a cell detachment solution (Accumax, Innovative Cell Technologies, San Diego, California) and counted with an automated cell counter (Nucleocounter NC100, Mandel Scientific, Guelph, Ontario). Population doubling times were calculated from passage 2 through passage 5 (AT-MSC, n = 8; BM-MSC, n = 6). For all the following experiments, MSC from passages 3–6 were used for this study.

### Immunophenotyping

Canine MSC (n≥3 for each cell source) were analyzed for surface marker expression using the Accuri C6 flow cytometer and software (BD, Mississauga, ON). Canine peripheral blood mononuclear cells (PBMC, n≥1) were used as controls. The antibodies used are listed in Table 1. All antibodies utilized were canine-specific except for MHC I (bovine) and CD73 (human), which were stated to cross-react with canine cells by the manufacturers and were validated with bovine and human PBMC respectively. Unstained samples of each MSC and PBMC were gated to determine surface marker expression of their stained counterparts.

**Table 1:** Cell surface marker list.

Antibody	Clone	Target	Host	Source
MHC I	H58A	Bovine	Mouse	Kingfisher Biotech
MHC II	YKIX334.2	Dog	Rat	AbD Serotec
CD4	YKIX302.9	Dog	Rat	eBioscence
CD8	YCATE55.9	Dog	Rat	AbD Serotec
CD14	TüK4	Dog	Mouse	ThermoFisher
CD29	MEM-101A	Dog	Mouse	ThermoFisher
CD34	1H6	Dog	Mouse	AbD Serotec
CD44	YKIX337.8.7	Dog	Rat	AbD Serotec
CD45	YKIX716.13	Dog	Rat	AbD Serotec
CD73	7G2	Human	Mouse	ThermoFisher
CD90	YKIX337.217	Dog	Rat	eBioscence

### Global DNA methylation quantification

Genomic DNA was isolated from AT- and BM-MSC samples (AT-MSC, n = 6; BM-MSC, n = 6) using a column purification system (Quick-gDNA MiniPrep, Zymo Research, Irvine, California). Global DNA methylation levels were quantified with a 5-methylcytosine ELISA kit according to the manufacturer's instructions (Zymo Research, Irvine, California).

### Trilineage differentiation

Trilineage differentiation (AT-MSC, n = 6; BM-MSC, n = 4) was performed as previously described except where indicated [18]. Briefly, for adipogenesis and osteogenesis, cells were cultured for 14 days with either EM as described above or induction medium. Adipogenesis induction medium consisted of low-glucose DMEM with 1 μM dexamethasone (Sigma-Aldrich, St. Louis,Missouri), 0.5 mM 3-isobutyl-1-methyl-xanthine (Sigma-Aldrich, St. Louis, Missouri), 10 μg/mL recombinant human (rh) insulin (Sigma-Aldrich, St. Louis, Missouri), 0.2 mM indomethacin (Sigma-Aldrich, St. Louis, Missouri), 15% rabbit serum (Sigma-Aldrich, St. Louis, Missouri), 1% L-glutamine, and 1% antibiotic antimycotic solution (ABAM, Sigma-Aldrich, St. Louis, Missouri). Osteogenesis induction medium consisted of low-glucose DMEM with 0.1 μM dexamethasone, 10 mM glycerol 2-phosphate, 0.05 mM ascorbic acid, 10% FBS, 1% L-glutamine, and 1% ABAM. For chondrogenesis, 250,000 cells were pelleted in a 96-well plate and cultured for 21 days in high-glucose DMEM (Lonza, Walkersville, Maryland), 0.1 μM dexamethasone, 0.1 mg/mL ascorbic acid (Sigma-Aldrich, St. Louis, Missouri), 10 ng/mL TGF-β3 (R&D Systems, Minneapolis, Minnesota), 200 mM Glutamax (Life Technologies, Grand Island, New York), 10 mg proline (Sigma-Aldrich, St. Louis, Missouri), 40 μg/mL ascorbic acid, 100 mM sodium pyruvate (Life Technologies, Grand Island, New York), 1% Insulin-Transferrin-Selenium (Life Technologies, Grand Island, New York), 1% L-glutamine, and 1% ABAM. To promote better chondrogenesis, 0, 50, 100, or 200 ng/mL bone morphogenic protein 2 (BMP-2) was added to the media.

Adipogenesis and osteogenesis samples were stained with Oil Red O and Alizarin Red S stains (Sigma-Aldrich, St. Louis, Missouri) respectively. Chondrogenesis samples were histologically evaluated with toluidine blue staining for glycosaminoglycan content and hematoxylin and eosin staining for general pellet structure as previously reported [36]. Adipogenic, osteogenic, and chondrogenic mRNA transcript abundance was analyzed by RT-qPCR using the primers listed in Table 2. cDNA was synthesized from 500 ng RNA using the High Capacity cDNA Reverse Transcription Kit (Life Technologies, Grand Island, New York) using manufacturers' instructions. PCR reactions were performed using the PerfeCta SYBR Green FastMix, ROX (Quanta BioScience, Gaithersburg, Maryland) with the Applied Biosystems 7300 Real Time PCR system. Data were analyzed using the 2^-ΔΔCT^ method. Gene expression data is presented as the induction medium-treated cultures relative to the expansion medium-treated control cultures with GAPDH used as reference gene.

**Table 2:** Oligonucleotide primer list.

Gene	Forward primer (5' to 3')	Reverse primer (5' to 3')	Reference
CEBPA	AGTCAAGAAGTCGGTGGACAAG	GCGGTCATTGTCACTGGTGAG	[11]
FABP4	ATCAGTGTAAACGGGGATGTG	GACTTTTCTGTCATCCGCAGTA	[11]
Leptin	CTATCTGTCCTGTGTTGAAGCTG	GTGTGTGAAATGTCATTGATCCTG	[11]
LPL	ACACATTCACAAGAGGGTCACC	CTCTGCAATCACACGGATGGC	[11]
PPARγ2	ACACGATGCTGGCGTCCTTGATG	TGGCTCCATGAAGTCACCAAAGG	[11]
Col1A1	GTAGACACCACCCTCAAGAGC	TTCCAGTCGGAGTGGCACATC	[11]
Runx2	AACCCACGAATGCACTATCCA	GGGACATGCCTGAGGTGACT	[37]
Osteopontin	GCACCTCTGACAGGGACAGCC	AGTGCTTGCGGCCCTTGGTT	
ALP	CCAACCTCCTGCCAACAAAAT	CTCTCATCTTTCCGAGCTCACA	
Sox9	TCCATCCCGCAGACGCACAG	GGATCATCGCGGCCACCCTT	
Col10A	AGTAACAGGAATGCCGATGTC	TCTTGGGTCATAATGCTGTTG	[11]
Aggrecan	GGGCTGGAAGCGTCATCAGT	AGGCTGAGGTGCCACCACTC	
Comp	GTGGTGGACAAGATTGATGTG	CACCCAGTTGGGATCTATCTG	[11]
GAPDH	TGTCCCCACCCCCAATGTATC	CTCCGATGCCTGCTTCACTACCTT	[38]

### Immunomodulatory properties

Whole blood was obtained from the jugular vein of dogs with an 18-gauge needle attached to a 450 mL blood collection bag (Fenwal, Baxter, Deerfield, Illinois). PBMC were isolated using a density gradient media (Ficoll-Paque Plus, GE Healthcare, Mississauga, Ontario). In a 50 mL tube, 7.5 mL of Ficoll-Paque Plus was added to the bottom. A 10 mL 1:1 mix of whole blood and PBS was then added on top and spun at 400 *g* for 20 min with acceleration set to 1 and deceleration set to 0. The mononuclear layers were pooled and washed repeatedly with PBS before resuspension in EM. PBMC were counted and frozen in EM with 10% DMSO (Sigma-Aldrich, St. Louis, Missouri) until use in the lymphocyte proliferation assays.

AT- and BM-MSC (AT-MSC, n = 4; BM-MSC, n = 4) were thawed and seeded 7 days prior to coculture with PBMC. On day 5, 4 treatments of each MSC were designated: 1) 200 ng/mL recombinant canine interferon-gamma (IFN-γ, Kingfisher Biotech, Saint Paul, Minnesota, Cat# RP0271D-025) added, 2) 50 ng/mL recombinant canine tumour necrosis factor-alpha (TNF-α, R&D Systems, Minneapolis, Minnesota, Cat#1507-CT-025) added, 3) both IFN-γ and TNF-α added, and 4) neither IFN-γ nor TNF-α added. Lymphocyte reaction plates (48 well) were set up on day 7. PBMC were seeded at 500,000 cells per well and stimulated with 5 μg/mL concanavalin A. MSC were irradiated with 20 Gγ and seeded at 50,000 cells per well. Corresponding MSC treatments were continued accordingly in the reaction plates. After 72 hours, 5-ethynyl-2'-deoxyuridine (EdU, a modified thymidine analogue) was added at a concentration of 10 μM and left for 24 hours before cells were collected and processed according to manufacturer's directions (Click-iT Plus EdU Flow Cytometry Assay Kit, Fisher Scientific, Ottawa, Ontario). EdU is incorporated during DNA synthesis and is used to quantify newly-synthesized DNA. Staining was completed the following day with Alexa Fluor 647 picolyl azide and analyzed using the Accuri C6 flow cytometer and software.

### Data analysis

Results were modelled as multi-factor factorials in a randomized complete block design treating dog as a blocking factor. Least squares means were determined. Log transformation of data was performed where necessary and back-transformed for readability. We tested residuals for normality and plotted them against the predicted values and factors to assess ANOVA assumptions and to look for unequal variance. We found that data were normal except for outliers in the adipogenesis data, but no outliers were removed. For the gene expression data, least squares means were converted to fold-difference by using 2^−*ΔΔCT*^. Data are presented as mean ± confidence interval with statistical difference assessed at P<0.05. All data analysis was performed using R statistical software (version 3.2.3, The R Foundation for Statistical Computing, Vienna, Austria).

## Results

### MSC isolation

As reported [18], our criteria for isolation success was based not only on colony formation, but also the ability to expand to a minimum 5 million cells. Accordingly, 8/8 AT-MSC and only 6/8 BM-MSC met these isolation criteria.

### MSC proliferation

**Fig 1:**
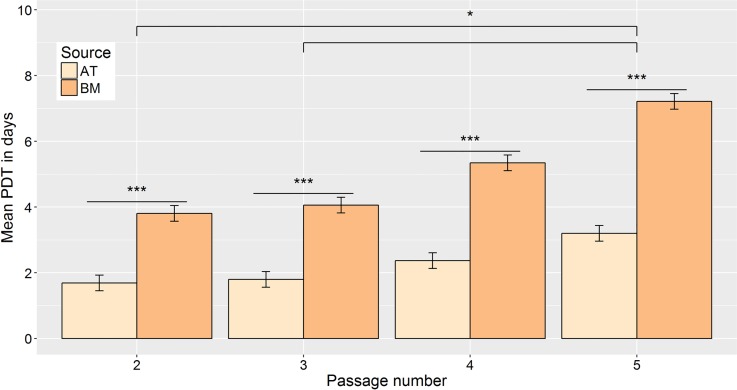
Adipose tissue (AT) derived mesenchymal stromal cells (MSC) proliferate faster than those derived from bone marrow (BM). Population doubling time of canine AT- and BM-derived MSC from passage 2 to 5. (*P<0.05, ***P<0.001; error bars = CI.)

AT-MSC proliferated faster than BM-MSC with significantly lower doubling times (P < 0.001) at all passages (P) between 2 and 5 (Fig 1). Proliferation rate also decreased with increasing passage for MSC from both cell sources as significant differences were found between both P2 and P5 (P = 0.02) and P3 and P5 (P = 0.02). Mean (± 95% confidence interval) doubling time in days were P2: (AT)1.72 ± 0.23, (BM)3.57 ± 0.23; P3: (AT)1.75 ± 0.23, (BM)3.62 ± 0.23; P4: (AT)2.30 ± 0.23, (BM)4.75 ± 0.23; P5: (AT)3.28 ± 0.23, (BM)6.77 ± 0.23.

### Immunophenotyping

Very similar cell surface molecule expression profiles were detected between AT- and BM-MSC (Table 3). MSC from both sources were highly positive for CD90, CD44, CD29, and MHC I while negative for CD45 and MHC II. Moderate expression of CD73, CD8 and mild expression of CD14 was also found in both cell types. The only differences seen were in the expression of CD4 (AT: moderate, BM: mild) and CD34 (AT: mild, BM: negative).

**Table 3:** Surface marker expression of canine adipose tissue (AT)-, bone marrow (BM)-derived mesenchymal stromal cells (MSC), and peripheral blood mononuclear cells (PBMC).

Surface marker	AT-MSC	SD	BM-MSC	SD	PBMC
MHC I	97.6	1.8	98.5	1.6	95.4
MHC II	4.7	1.7	1.5	0.3	99.0
CD4	48.0	3.5	19.9	9.7	84.9
CD8	55.3	11.5	58.6	18.6	83.5
CD14	7.1	2.9	7.6	1.0	94.8
CD29	81.8	12.2	83.6	18.4	38.0
CD34	18.6	3.4	3.6	1.5	92.7
CD44	100.0	0.0	99.8	0.2	100.0
CD45	1.5	0.3	1.5	0.4	99.2
CD73	63.2	5.7	59.9	12.5	97.7
CD90	99.6	0.3	89.0	7.5	89.4

### Global DNA methylation quantification

**Fig 2:**
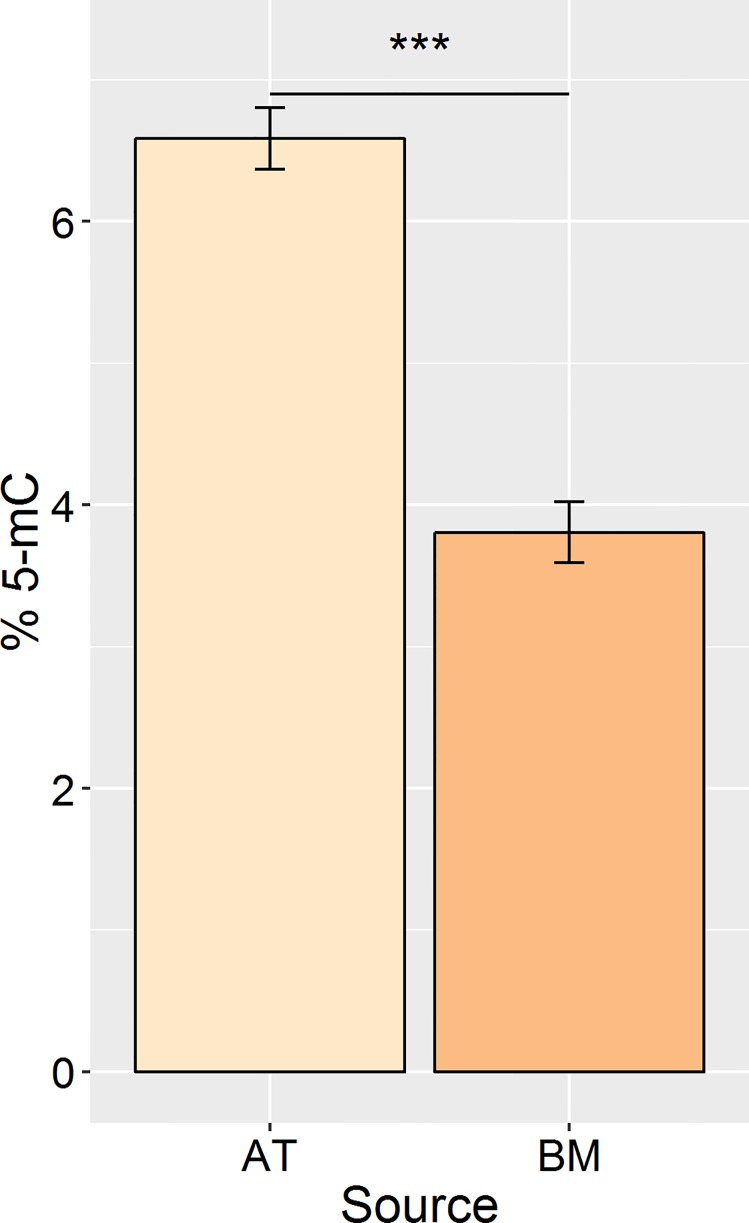
Percent 5-mC detected in genomic DNA from canine adipose- and bone marrow-derived mesenchymal stromal cells. (***P<0.001; error bars = CI.)

AT-MSC showed significantly higher (P<0.001) genome-wide DNA methylation levels (6.59 ± 0.52%) than BM-MSC (3.81 ± 0.30%) (Fig 2).

### Trilineage differentiation

**Fig 3:**
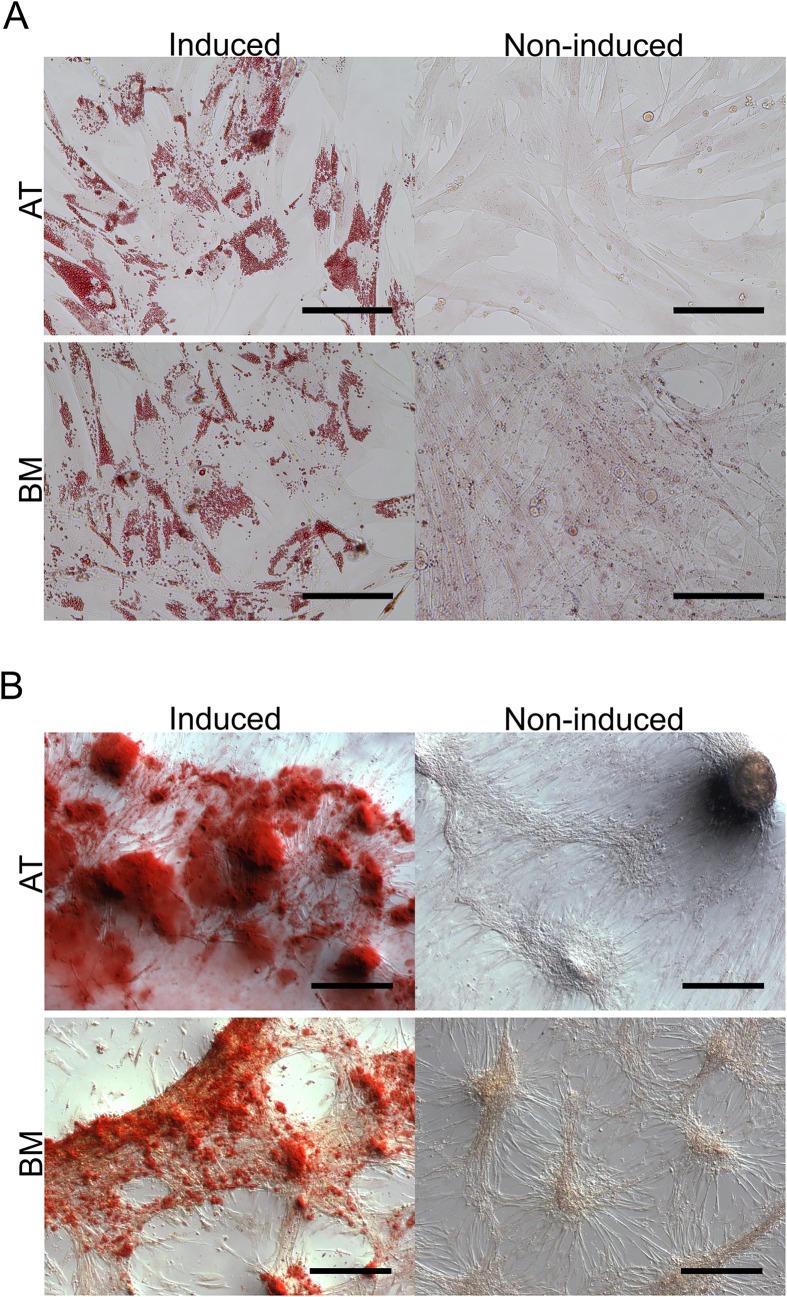
Adipogenic and osteogenic induction of AT- and BM-derived canine MSC. (A) Adipogenic potential of both canine adipose tissue (AT)- and bone marrow (BM)-derived mesenchymal stromal cells was indicated with positive Oil Red O staining after 14 days in induction medium. Control samples were negative for Oil Red O staining. Scale bars = 100 μm. (B) Osteogenic potential of both canine adipose tissue (AT)- and bone marrow (BM)-derived mesenchymal stromal cells was indicated with positive Alizarin Red S staining after 14 days in induction medium. Control samples were negative for Alizarin Red S staining. Images were adjusted for brightness and contrast. Scale bars = 200 μm.

**Fig 4:**
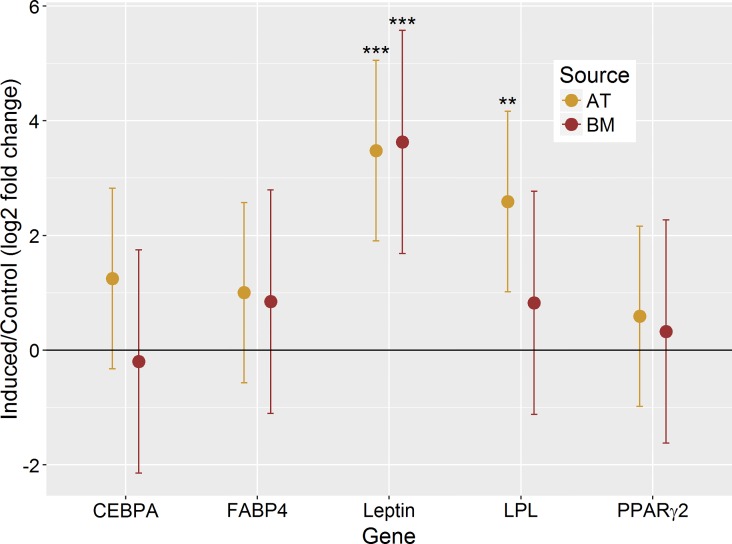
Upregulation of adipogenesis markers leptin and lipoprotein lipase (LPL). Difference in adipogenesis marker expression of canine adipose tissue- and bone marrow-derived mesenchymal stromal cells after 14 days in induction medium. (*P<0.05, **P<0.01, ***P<0.001; error bars = 95% CI.)

**Fig 5:**
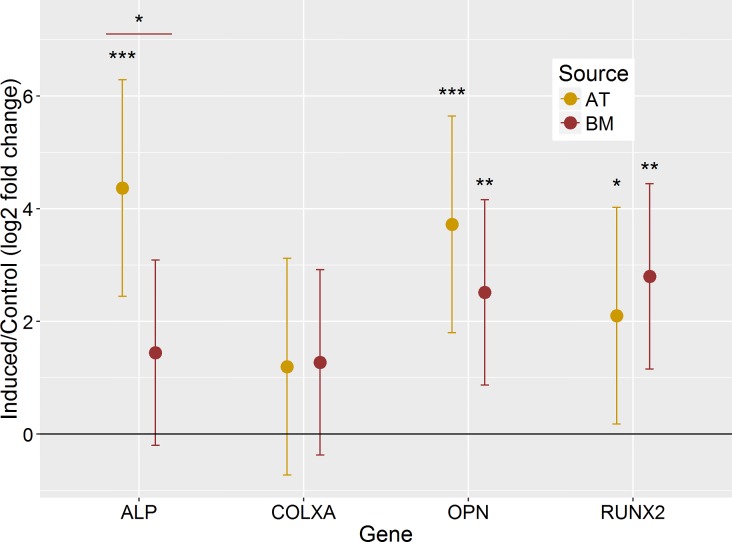
Upregulation of osteogenesis markers alkaline phosphatase (ALP), osteopontin (OPN), and Runt-related transcription factor 2 (RUNX2). Difference in osteogenesis marker expression of canine adipose tissue- and bone marrow-derived mesenchymal stromal cells after 14 days in induction medium. (*P<0.05, **P<0.01, ***P<0.001; error bars = 95% CI.)

**Fig 6:**
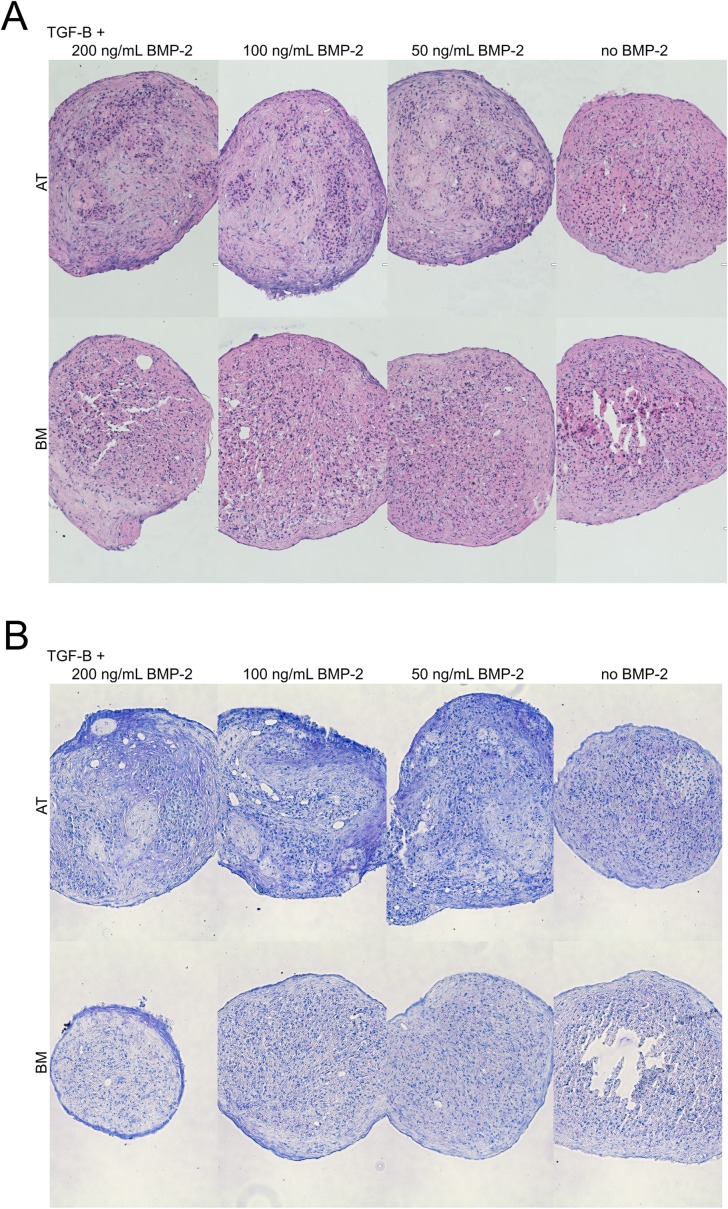
Poor chondrogenic potential of both canine adipose tissue (AT)- and bone marrow (BM)-derived mesenchymal stromal cells. Induction time was 21 days in medium containing 10 ng/mL transforming growth factor beta 3 (TGF-β) and between 0 and 200 ng/mL bone morphogenic protein 2 (BMP-2). Samples stained with (A) hematoxylin and eosin and (B) toluidine blue. Images were adjusted for brightness and contrast. Scale bars = 100 μm.

After an induction period of 14 days, both AT- and BM-MSC stained positive for adipogenesis (Fig 3A) and osteogenesis (Fig 3B). Adipogenic mRNA transcript abundance (Fig 4) of leptin was upregulated in AT-MSC (11.15-fold, P<0.001) and BM-MSC (12.40-fold, P<0.001) and lipoprotein lipase (LPL) was upregulated in AT-MSC only (6.02-fold, P = 0.002). Osteogenic mRNA levels (Fig 5) were upregulated for osteopontin (OPN) in AT-MSC (13.21-fold, P<0.001) and BM-MSC (5.73-fold, P = 0.004) and for Runt-related transcription factor 2 (RUNX2) in AT-MSC (4.30-fold, P = 0.03) and BM-MSC (6.96-fold, P = 0.002). A significant difference was discovered (P = 0.03) between AT- and BM-MSC for alkaline phosphatase (ALP) mRNA with upregulation found only in AT-MSC (20.63-fold, P<0.001). Chondrogenesis was unsuccessful (Fig 6) after a 21 day induction period regardless of the concentration of BMP-2 added. However, AT-MSC are BMP-sensitive as noted by increased Toluidine Blue staining and more heterogeneous tissue formation compared to TGF-β3 alone as well as BMP supplemented BM-MSC.

### Immunomodulatory properties

**Fig 7:**
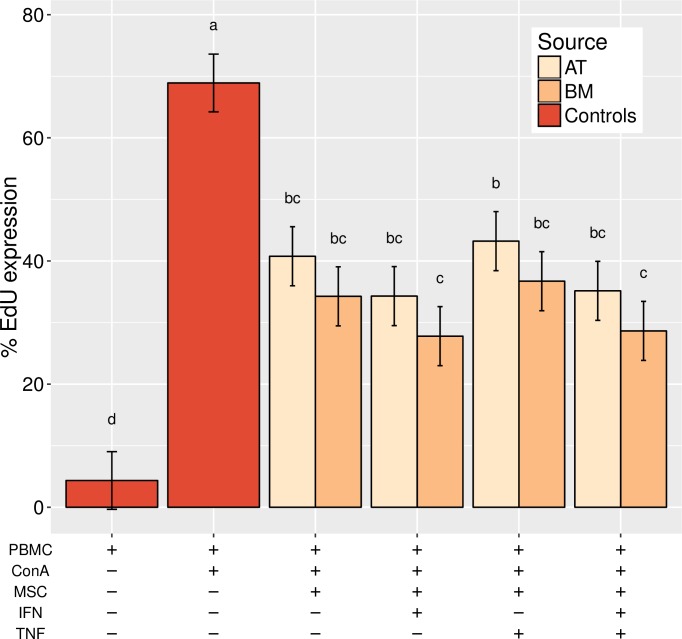
Canine MSC inhibit T-cell proliferation. Concanavalin A-stimulated peripheral blood mononuclear cells (PBMC) were cocultured with adipose tissue- or bone marrow-derived MSC treated with interferon-gamma, tumour necrosis factor-alpha, both, or neither. Stimulated and unstimulated PBMC were used as controls.

Lymphocyte proliferation assays were used to assess the lymphocyte-suppressive capacity of different canine MSC populations. AT- and BM-MSC equally suppressed stimulated PBMC proliferation when compared with stimulated PBMC alone (Fig 7). Priming the MSC with treatments of IFN-γ, TNF-α, or both had no effect on MSC immunomodulatory capacity.

## Discussion

This is the first study to compare the immunomodulatory capacities of canine AT- and BM-MSC in addition to evaluating their general characterization and differentiation potentials. We found that both sources of MSC had proficient immunosuppressive properties. In characterizing AT- and BM-MSC, we found no profound differences between the cell types except for the significantly higher expansion rate of AT-MSC, which has been previously reported [39]. Faster proliferation along with the potential for a less invasive method of their procurement makes them the preferred source for canine MSC.

We cocultured PBMC stimulated with Con-A with irradiated AT- or BM-MSC in order to determine whether they could suppress lymphocyte proliferation. We tested MSC cultured with and without proinflammatory factors IFN-γ, TNF-α, or both for 3 days leading up to, plus the 4 days of, coculture with PBMC. While all treatment groups successfully suppressed PBMC proliferation, no treatment significantly outperformed any other within each source group (Fig 7). It is possible that a larger sample size would discern greater differences trending toward BM-MSC as PBMC proliferation is consistently lower in these wells across treatments.

If differences in immunomodulatory capacity were to emerge, the question of whether differences in surface marker expression might correlate with a more potent immunosuppressive phenotype becomes an interesting one. Our results are in agreement with the canine literature for those surface markers that show consistent expression across several studies [6,10,15,35,36,39–43], in particular, positive CD90 and CD44 and negative CD45 expression. AT-MSC showed moderately higher expression of CD34 (18.6% ± 3.4) than BM-MSC (3.6% ± 1.5) and CD4 (48.0% ± 1.7 versus 19.9 ± 9.7). All other markers fell within the same ranges of expression as seen in Table 3. It has been recently reported that fat and bone marrow harvest sites do have some influence on surface marker expression [14], and likely played a role here as well.

Our first attempt at chondrogenesis of canine MSC [18] was poorly demonstrated after 21 days in the induction medium we use routinely with equine MSC [37,44]. To enhance our induction medium, we added BMP2 at different concentrations based on several reports showing it was a potent driver of MSC chondrogenesis [45–49]. Unfortunately, in spite of these efforts, chondrogenesis did not improve (Fig 6). Pellets generally appeared necrotic with no evidence of lacunae formation across all samples. AT-MSC were BMP sensitive as evidence by altered pellet morphology and Toluidine Blue staining pattern. BMPs therefore remain candidates for aiding the chondrogenic differentiation, but more work is needed to determine their temporal and co-induction molecular interplays. It should be noted that canine chondrogenesis has not been robustly demonstrated in the literature as has been noted by others [10]. Until an effective induction protocol is found, it appears that *in vitro* MSC chondrogenic differentiation is limited in the dog compared to other species.

Adipogenic potential was demonstrated with induced cells rich with lipid droplets stained with Oil Red O (Fig 3A). Histological data was supported by gene expression analysis showing upregulation of adipogenesis markers leptin in both AT- and BM-MSC and LPL in the AT-MSC samples (Fig 4). Likewise, osteogenic potential was also demonstrated with evident mineralization stained with Alizarin Red S supported by upregulation of osteogenesis markers OPN and RUNX2 in both AT- and BM-MSC and ALP in AT-MSC. It was thought that the reduced global DNA methylation levels of BM-MSC (Fig 2) might provide the cells stronger differentiation potential [50,51]. However, at least with the three lineages induced, global DNA methylation levels had little effect. It would be interesting to examine the ability of these cells to differentiate outside the trilineage cell fates.

Ultimately, there were few differences detected between AT- and BM-MSC with regard to immunophenotyping, differentiation potential, or immunomodulatory capacity. Major differences between the sources of MSC were only found in DNA methylation levels and proliferation doubling time. This may seem counterintuitive, but this simple measure of global methylation accounts neither for specific patterns of DNA methylation nor other factors affecting gene expression like histone modification. While the difference in DNA methylation appears to have no detectable effect on differentiation potential, a higher rate of proliferation provides a key advantage to AT-MSC.

Regardless of cell source, the significant *in vitro* suppression of mononuclear cells warrants *in vivo* investigation of canine AT- and BM-MSC efficacy in modulating the immune system of inflammation-based conditions. As for their progenitor side, new protocols for chondrogenesis will need to be developed if canine MSC are to serve as chondroprogenitor cells. Failing that, other canine cells with chondrogenic potential should also be considered.

## Supporting Information

S1 File Data for proliferation (population doubling time). (CSV)

S2 File Data for immunophenotyping. (CSV)

S3 File Data for global DNA methylation quantification. (CSV)

S4 File Data for adipogenesis marker gene expession. (CSV)

S5 File Data for osteogenesis marker gene expression. (CSV)

S6 File Data for lymphocyte proliferation assays. (CSV)
